# An innovative approach to enhancing the strength and durability of recycled aggregate concrete through fly ash-silica fume coating and rice husk ash supplementation

**DOI:** 10.1038/s41598-025-18138-z

**Published:** 2025-09-25

**Authors:** Ahmed A. Alawi Al-Naghi, Tariq Ali, Inamullah Inam, Muhammad Zeeshan Qureshi, Nabil Ben Kahla, Nejib Ghazouani

**Affiliations:** 1 Civil Engineering Department, University of Ha’il, Ha’il, 55476 Saudi Arabia; 2 Department of Civil Engineering, Swedish College of Engineering and Technology, Wah, 47080 Pakistan; 3 Department of Civil Engineering, Engineering Faculty, Laghman University, Mehtarlam, Afghanistan; 4 Department of Civil Engineering, University of Engineering and Technology, Taxila, Pakistan; 5 Department of Civil Engineering, College of Engineering, King Khalid University, PO Box 394, Abha, 61411 Kingdom of Saudi Arabia; 6 Center for Engineering and Technology Innovations, King Khalid University, Abha, 61421 Saudi Arabia; 7 Mining Research Center, Northern Border university, Arar, 73213 Saudi Arabia

**Keywords:** Recycled aggregate concrete, Slurry coating, Rice husk ash, Mechanical and durability properties, Engineering, Materials science

## Abstract

The building industry responded to the growing imperative to reduce the global ecological footprint by developing new inventive methods of utilizing waste materials. Concrete waste is one of the leading contributors to global waste streams and a great opportunity for sustainable reuse. Recent research has demonstrated its potential for structural applications in concrete, although its use for nonstructural component construction has been well established. This study introduces an innovative approach, combining the impact of S-FA (silica fume-fly ash) slurry coated recycled aggregates (RCA) and supplementary cementitious material (Rice husk) simultaneously, for the first time to be evaluated for structural performance. These findings present a major improvement in the reuse of concrete waste for structural applications by overcoming the constraints recognized by the mechanical characteristics of RCA. The study is based on an extensive experimental program which evaluated critical parameters such as compressive and tensile strength, water absorption and acid resistance. The incorporation of 100% treated RCA aggregates improved compressive strength by 23% compared to the control mix of non-treated 100% RCA aggregates. Similarly for the same mix acid resistance strength demonstrated a 7% increase while the Non-destructive test results revealed that the treatment enhanced RCA performance by providing improved results through ultrasonic pulse velocity (UPV) readings that increased by 19% compared to the control mix of non-treated RCA aggregates (100%).

## Introduction

The rapid urbanization and high contribution to the national economic development has led to the construction industry drastically growing, while this expansion has brought about these environmental challenges, depletion of natural resources, the production of massive quantities of C&D (construction and demolition) waste, and widespread land poisoning^1–3^. It is estimated in some countries that the production of C&D waste is around 1.8 billion tons annually, which is growing at a rate of 5–10% per year and this accounts for a substantial portion of the overall waste generated worldwide^2,3^. Sustainable solutions to these challenges are the utilization of RCA (recycled concrete aggregate) made from C&D waste, generated through screening, crushing, and grading processes. The employment of these waste materials helps in efficient waste management, helps in reducing resource depletion caused due to over-exploitation and minimizing environmental issues accompanying C&D waste^4,5^. Generally, recycled coarse aggregates have an irregular shape, containing some residual mortar on their surfaces and most of the time they have lower physical properties than natural coarse aggregates. Due to variations in its composition, recycled concrete aggregates have multiple weaker transition zones and are less durable than concrete including NA (natural aggregates). Strength losses and decreased durability in recycled aggregates can be attributed to the porous nature of the attached mortar. Both the mechanical and durability performance of recycled aggregate concrete are significantly compromised, so these limitations negatively affect the feasibility of RAC for structural applications^6–9^.

In order to improve the RCA porous structure, techniques like slurry coating or surface coating using the pozzolanic material offer a sustainable and cost-effective solution^10,11^. This approach enables the incorporation of several cementitious by-products associated with industrial developments including, FA (fly ash) which originates from power station furnaces, blast-furnace slag that originates from the blast furnaces, and SF (silica fume) is associated with electric arc furnaces^12^. Tam and Tam^13^ revealed that the use of SF during the concrete mixing process efficiently blocks the pores on the RCA surface. This produces a more durable coating around the RCA particles, resulting in recycled aggregate concrete with enhanced strength. The research conducted by Li et al.^14^ and Kong et al.^15^ on the effects of a novel mixing and coating process using pozzolanic materials on recycled aggregate concrete (RAC), findings reveal that this technique enhanced the compressive strength and flexural strengths of RAC along with densified the ITZ (interfacial transition zone). An approach to surface treatment was suggested by Bui et al.^16^ that involved immersing RCA in a sodium silicate solution and then coating its surface with silica fume. This method improves the characteristics of RAC and the quality of RCA. Shaban^11^ analyzed the effects of utilizing pozzolan slurries in recycled coarse aggregates (RCA), using silica fume, nano-silica fume and fly ash. According to the findings of this study, water absorption of RCA was decreased by 50–55%, and the density of particle was enhanced by 10–11%. These enhancements in the properties of the RCA were associated with the ability to soak in pozzolan slurry to improve the strength of the weak mortar attached to the RCA surface. This improvement occurs since the pozzolanic materials interact with the calcium hydroxide (C–H) available in the attached mortar which leads to further formation of C–S–H gel which in turn improves the roughness of the aggregate surface^11,17^.

Several studies have conducted extensive research on the use of recycled aggregates (RA) and RHA (rice husk ash) in concrete. Koushkbaghi et al.^18^ reveal that the incorporation of 20% of cement with RHA in RAC improves late-stage hydration reactions and leads to higher mechanical values and durability as compared to plain RHA concrete. Padhi et al.^19^ also found that using 100% recyced Coarse Aggregates and 10–15% RHA was effective for engineering applications. Similarly, Rattanachu et al.^20^, found that a 20% replacement of RHA was responsible for an enhanced pozzolanic reaction that helped in increasing the strength of RAC. Chatveera, B., & Lertwattanaruk, P. (2011) demonstrated that the incorporation of BRHA (black rice husk ash) significantly improved the durability of the concrete exposed to hydrochloric and sulfuric acid conditions besides reducing AS (autogenous shrinkage) as the percentage of BRHA was added^21^. Furthermore, short-term accelerative corrosion tests by Ferraro and Nanni,2012^22^ Indicated that the incorporation of WRHA (white rice husk ash) enhanced the corrosion protection of steel bars use in concrete. This paper focuses on a dual-treatment method, and it applies a combination of mechanical grinding along with coating of recycled concrete aggregates with fly ash-silica fume (S-FA) slurry and adding rice husk ash (RHA) as a supplement. The work develops on the established mechanisms in microstructure^23^ to specifically test the effects of the treatment on important engineering properties through experimentation, and then complementary microstructural understanding based on the validated literature^23^. This work focuses on designing a holistic RCA enhancement methodology in terms of meeting mechanical performance and structural durability demands.

## Novelty of research

This research paper enhances RCA treatment technology by integrating mechanical grinding, S-FA slurry adhesion and RHA supplementation-a combination that has not been attempted in the literature. The main differentiators are shown in Table 1.

**Table 1:** Comparative analysis of prior vs. this study.

Aspect	Previous Research	This Research	Advancement
**Treatment Methods**	Single treatments: slurry coating [11] or mechanical processing [13]	Combined mechanical grinding + S-FA slurry coating	Dual-action approach improves ITZ densification and surface roughness
**SCM Utilization**	RHA *or* S-FA used separately [19], [20]	RHA (15%) + S-FA slurry (40%) simultaneously	Synergistic pozzolanic effects enhance strength and durability
**Durability**	Primarily compressive strength improvement [15]	Comprehensive: acid resistance, water absorption, UPV, *and* mechanical tests	Significant performance evaluation for structural applications

## Materials and methods

In the manufacturing process of all blends Type I OPC (ordinary Portland cement) was used as the primary binder. In Pakistan, Maple Leaf cement brand was used in this study which is readily accessible and commonly locally available. This cement meets all the criteria provided by ASTM C150 standards. Silica fume (SF) and F-class fly ash (FA) were purchased from a local supplier, Imporient Chemicals, located in Punjab, Pakistan. The chemical characteristics of cement, SF and FA are listed in Table 2 while Table 3 shows the physical characteristics. Fine aggregates (sand) use in this research were sourced from Lawrencepur, Pakistan. Coarse aggregate was used, sourced from Margalla Pakistan, both aggregates meet the specification of ASTM C 33 standard. Table 4 presents the physical characteristics of coarse aggregate and fine aggregate.

The level of RCA replacement of 0-100% was selected to assess the recycled concrete aggregate performance with different levels of incorporation since the results provided by previous research paper^11^ indicate that replacing 50% of the concrete with RCA has acceptable outcomes, whereas the higher levels (75–100%) ill indicate the barriers and the necessity of treatment methods to enhance concrete performance. Likewise, 15% o RHA content was chosen due to the substantiation in the literature that it is effective to enhance pozzolanic reaction and increase the durability of concrete, which is confirmed by the study of^18,19^ that concludes that RHA content of 10–20% is optimal o enhance strength and durability of concrete mixtures.

**Table 2:** Chemical characteristics of OPC, SF and FA.

Elements	OPC	Silica fume	Fly ash
SiO_2_	18.52	95.33	47.36
CaO	63.68	1.5	6
Al_2_O_3_	6.41	1.48	32
Na_2_O	0.21	0.10	0.49
Fe_2_O_3_	6.05	0.35	8.02
SO_3_	1.82	0.15	0.55

**Table 3:** Physical characteristics of OPC and SF.

Properties	OPC	Silica fume
Sp. surface area	341 m^2^/kg	15 m^2^/gm
Setting time (Initial)	68 min	---
Setting time (final)	260 min	---
Sp. gravity	3.13	2.20 g/cm^3^
Normal Consistency	30.4%	---
Density	1440 kg/m^3^	2200 kg/m^3^
Mean particle size	12 μm	230 nm

**Table 4:** Physical characteristics of aggregates.

Properties	Crush	Sand
Normal Maximum Size	20 mm	5 mm
Fineness modulus	----	2.92
Sp. Gravity	2.65	2.82
Dry-rodded unit weight	----	1560 kg/m^3^
Water absorption	0.80%	1.30%
Bulk Density	1645 kg/m^3^	1455 kg/m^3^
Moisture Content	0.15%	0.32%

### Experimental program

**Fig. 1:**
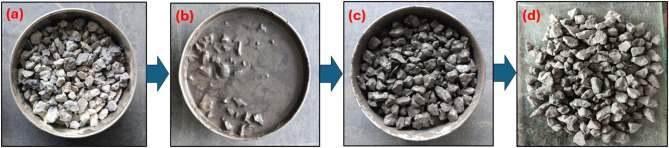
RA Treatment process (a) RA (b) Soaked in FA and SF slurry (c) Wet condition (d) Dry condition.

**Fig. 2:**
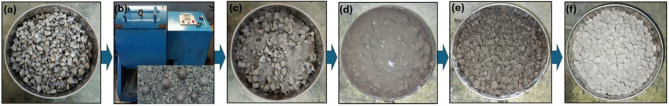
Mechanically treated process (**a**) RA (**b**) Los Angeles abrasion apparatus (**c**) Grinded RA (**d**) Soaked in Slurry (**e**) Wet condition (**d**) Dry condition.

This section consists of three groups: Group 1 incorporated an untreated RCA into the concrete mixture while Group 2 used RCA which has been treated with slurry (SF and FA) to improve the concrete ITZ through filling of the pores present on the RCA surface shown in Fig. 1. For Group 3, the RCA was mechanically grounded using a Los Angeles abrasion apparatus to increase the roughness of the surface and treated with the same slurry to improve the bonding and durability shown in Fig. 2. The RCA was soaked in Slurry for 4 h. The quantity of the material for slurry is mentioned in Table 5. The dosage quantity for treated slurry is determined by previous research^11^. The test was performed after curing periods (7, 28, and 90 days) to assess the mechanical and durability properties of concrete. To evaluate the resistance of the tested sample to acid attack, the samples were soaked in 5% H_2_SO_4_ solution for 1 month and 3 months respectively. The specimens were cured in water tanks at a temperature of 23 °C ± 2 °C and a relative humidity of 30% ± 5%. The summary of tests conducted, including specimen type, size and their corresponding standards are listed in Table 6.

**Table 5:** Slurry mix proportions for RCA treatment (per batch)^11^.

Materials	Values
Fly ash (40%)	200 kg
Silica fume (40%)	200 kg
Aggregate	1000 kg
Water	2000 kg
B/W (Binder to water ratio)	1:5

**Table 6:** Test details, specimen size and type with their corresponding standards.

Tests	Specimens		ASTM Standards
	Type	Size (mm)	
Compressive Strength	Cylinder	150 × 300	C-39
Tensile Strength			C-496
Acid Attack			C-1898
Water absorption			C-642
UPV			C597-16
RN			C-805

## Mix proportion

This study followed the ASTM C192/C192M standard while mixing the concrete. The OPC content was constant at 450 kg/m³. To enhance the pozzolanic activity of the mix 15%^24^ of OPC was replaced by RHA (67.5 kg/m³) a byproduct with a high content of silica that improves concrete strength and durability while reducing cement dependency, fine aggregates at 600 kg/m³ was used to ensure adequate particle packing and workability. RCA substituted NCA in proportions of 0%, 25%, 50%, 75% and 100%. A constant W/B (water-binder ratio) of 0.45 and 1% superplasticizer (5.17 kg/m³) was used for uniform mixing and strength development. The mix design of all groups is presented in Table 7.

**Table 7:** Mix design for all groups.

Mixes	OPC (kg/m^3^)	RHA 15% (kg/m^3^)	RCA (kg/m^3^)	NCA (kg/m^3^)	FA (kg/m^3^)	W/b (0.45)	SP 1% (kg/m^3^)
CON(G0-R0)	450	67.5	0	1000	600	232.8	5.17
GP1-R25	450	67.5	250	750	600	232.8	5.17
GP1-R50	450	67.5	500	500	600	232.8	5.17
GP1-R75	450	67.5	750	250	600	232.8	5.17
GP1-R100	450	67.5	1000	0	600	232.8	5.17
GP2-R25	450	67.5	0	1000	600	232.8	5.17
GP2-R50	450	67.5	250	750	600	232.8	5.17
GP2-R75	450	67.5	500	500	600	232.8	5.17
GP2-R100	450	67.5	750	250	600	232.8	5.17
GP3-R25	450	67.5	0	1000	600	232.8	5.17
GP3-R50	450	67.5	250	750	600	232.8	5.17
GP3-R75	450	67.5	500	500	600	232.8	5.17
GP3-R100	450	67.5	750	250	600	232.8	5.17

*GP1 (Untreated): No treatment applied to the recycled concrete aggregates (RCA). *GP2 (Slurry Coated): RCA treated with slurry (combination of fly ash and silica fume). *GP3 (Mechanically Ground + Slurry Coated): RCA subjected to mechanical grinding followed by slurry coating.

## Results and discussion

### Compressive strength

**Fig. 3:**
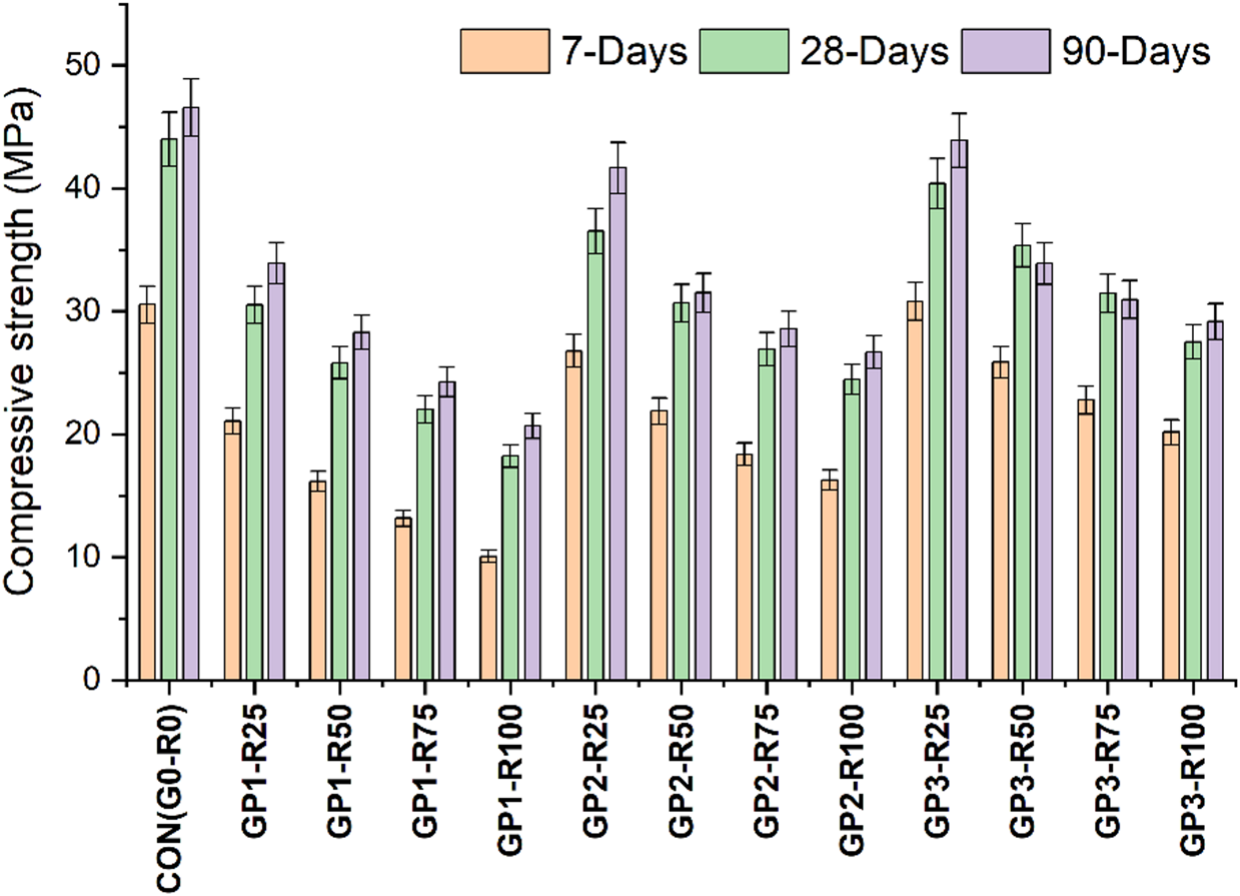
Compressive strength findings of different Groups.

The compressive strength illustrated in Fig. 3. The results reveal the impact of incorporating RCA on the mechanical properties of RAC, highlighting a consistent trend of decreased strength as the content of RCA increases. Concrete with NA only (CON-G0-R0) exhibits the highest strength at all curing ages (7,28 & 90 days), indicating that natural aggregates provide better mechanical properties that result in a stronger ITZ (interfacial transition zone) and a stronger concrete matrix. The control mix attains a strength of 30.53 MPa (7 days), 43.97 MPa (28 days), and 46.56 MPa (90 days). Furthermore, the results indicate that all mixes containing a portion of RCA display a dramatic decrease in compressive strength proportional to RCA substitute levels. The cause of this phenomenon is the inherent nature of RCA such as adhered mortar and higher porosity compared to natural aggregates. All these factors compromise the integrity of the interfacial transition zone (ITZ), the ITZ being vital to the mechanical strength of the concrete. Therefore, as the concrete continues to mature, its strength is certainly limited by these characteristics of RCA^25,26^. Group-1 (GP1) utilized untreated RCA, exhibited a significant reduction in strength at 7 days decline from 22.1 MPa for 25% RCA (GP1-R25) to 10.9 Mpa for 100% RCA (GP1-R100), demonstrating a sharp reduction in early strength as the RCA content increases. Similar patterns are obtained at 28 and 90 days, whereas the strength decreases from 31.5 MPa for 25% RCA (GP1-R25) to 19 MPa for 100% RCA (GP1-R100) and from 34.9 MPa to 21.5 MPa, respectively, for the same mixes. Consequently, these results indicate that while RCA offers environmental and economic benefits, it also restricts the mechanical performance of concrete at higher replacement levels.

Similarly, Group-2 (GP2) having the slurry-coated RCA exhibited slightly improved compressive strength in comparison with GP1. This indicates that RCA treatment can mitigate some of the adverse effects compared to RCA incorporation and contribute to better performance. The Compressive strength for GP2-R25 and GP2-R100 at 7 days is found to be 26.8 MPa and 16.3 MPa respectively. The strength after 28 days of the mix GP2-R25 and GP2-R100 increases to 36.5 MPa and 24.5 MPa respectively. At 90 days the concrete strength is found to be 41.7 MPa for GP2-R25 and 26.7 MPa for GP2-R100 mix. However, the overall trend still indicates a reduction in strength with raising RCA content, highlighting the inherent weakness of RCA and its persistent impact on the performance of concrete mix.

Likewise, Group-3 (GP3), which utilized RCA treated with a combination of mechanical grinding followed by FA and SF slurry coating achieved the highest compressive strength. This treatment technique shows the most effective approach for addressing RCA-related challenges and enhances the overall performance of RAC. The compressive strength of GP3-R25 is 30.8 MPa (7 days), 40.4 MPa (28 days), and 43.9 MPa (90 days). At higher replacement levels (like GP3-R100) compressive strength values are still higher than those of the similar mixes from the GP1 and GP2 group, 20.2 MPa at 7 days, 27.5 MPa at 28 days, and 29.2 MPa at 90 days. The results indicate that the limitations of RCA can be almost fully overcome with the implementation of combo-treated RCA and can be used for structural concrete.

This study compares the variations in compressive strength based on treatment groups. Group 1 (GP1), i.e. RCA without treatment, showed the least compressive strength proving the adversities implicated in RCA without treatment. However, Group 2 (GP2) exhibited a 13% enhancement in compressive strength for 100% RCA incorporation, highlighting the significance of slurry coating aggregate treatment. In addition, Group 3 (GP3), combined treatment (RCA: Mechanical grinding + Coating with slurry), produced the highest compressive strength of 21% for 100% RCA incorporation. Furthermore, GP3 development appears to provide the most suitable solution to RCA-related problems, pointing to the advantage of advanced treatment techniques in improving the RAC performance. The statistical values are presented in Table 8.

The reason for gaining strength with the RCA treatment in GP2 and GP3 is attributed to the treatment’s ability to address the high-water absorption of RCA. This treatment reduces the porosity of the RCA and improves ITZ in the concrete matrix^15^. The incorporation of silica-rich pozzolanic materials in recycled concrete aggregate (RCA) facilitates a pozzolanic reaction with free CH (calcium hydroxide) from the adhered mortar. This reaction creates a dense coating on RCA particles and fills internal pores with hydrated C-S-H gel that significantly increases the material’s density and durability, as well as improves structural integrity and reduces porosity and water absorption^23,27–29^.It is evident from previous research that using pozzolanic materials like silica fume and fly ash or a combination of both strengthens RCA by reacting with calcium hydroxide in the adhered mortar, forming C-S-H gel^11,15,30^.

A previous study indicated that RAC with TSMAs was most improved in terms of strength and rigidity for certain types of substitutions of RA. After 28 and 56 days of curing, TSMAs showed improvements of 19.50 and 19.58% at 25% RA substitution which shows the benefit of using TSMAs in the long term. Additionally, the strength with 100% replacement of RA increased by 16.16% in 28 days and 30% with RA (strength increased by 16.28%) at 28 days. These findings led to the results which show that TSMAs can increase RAC’s structural capacity and can be used in sustainable construction applications^13^. This is attributed to the property of ultrafine particle size and the high pozzolanic activity of silica fume that reduces the pore size of weak attached mortar of RCA. This process helps reduce the inherent porosity of the contained mortar and creates a very dense layer around the RCA particles. As it was applied, this strengthened coating enhances the aggregate’s structural integrity and performance in concrete mixtures^13^.

**Table 8:** Statistical values for compressive strength.

Curing days	Mean	Standard Deviation	SE of Mean
14-Days	21.06	6.33	1.75
28-Days	30.28	7.32	2.03
90-Days	32.30	7.66	2.12

### Tensile strength

**Fig. 4:**
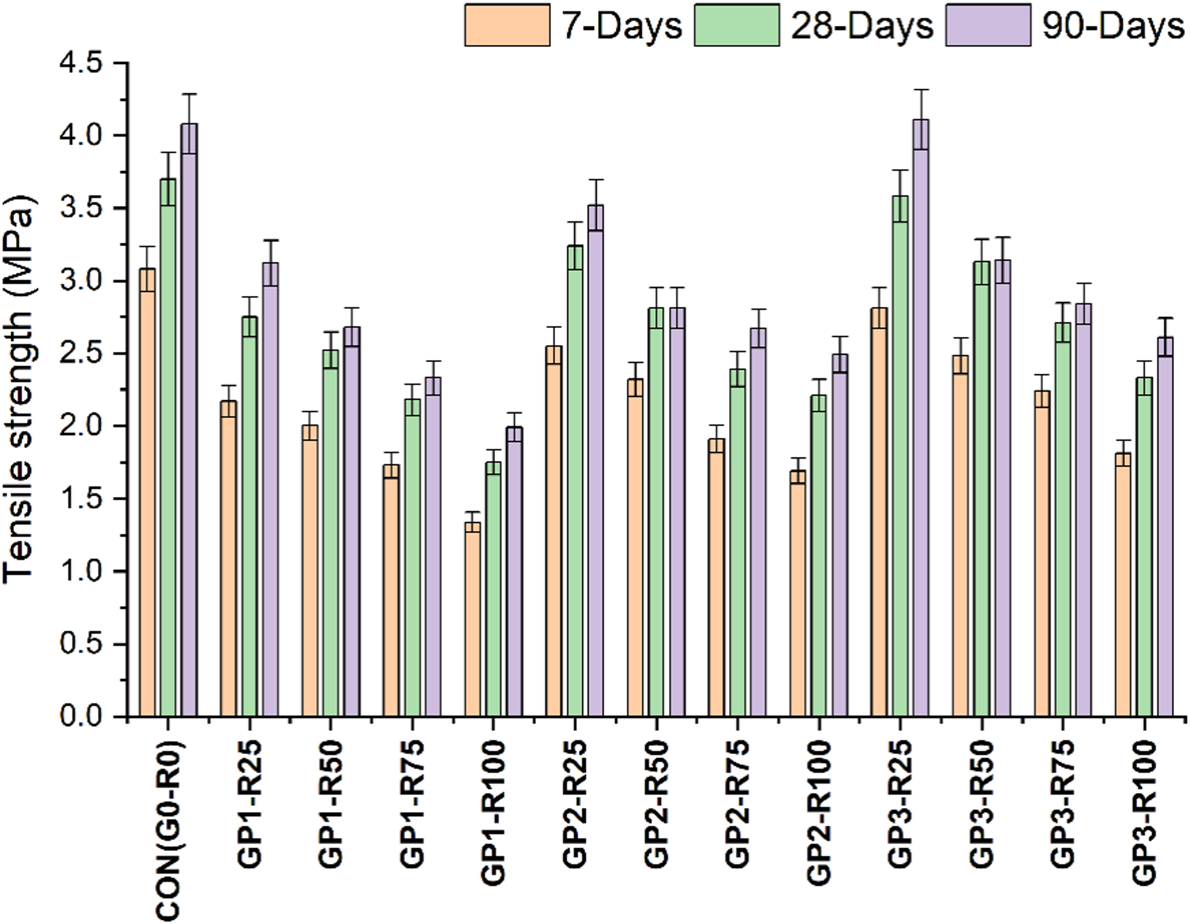
Tensile strength findings of different Groups.

The tensile strength calculated is shown in Fig. 4, revealing the development of strength over time and the impact of RCA on the mechanical properties of RAC. The data illustrates how the mechanical performance of RAC is affected by the incorporation of RCA; tensile strength is consistently declining as RCA proportion increases. The control mix attains a tensile strength of 3.08 Mpa (7 days), 3.70 MPa (28 days) and 4.06 MPa (90 days), which demonstrates the cumulative strength of conventional concrete.

In Group 1 (GP1), which utilizes untreated RCA, the tensile strength drops from 2.17 MPa for 25% RCA (GP1-R25) to 1.34 MPa for 100% RCA (GP1-R100) after 7 days, demonstrating a steep reduction in early tensile strength as the RCA content increases. Similar trends are seen at 28 and 90 days when the tensile strength decreases from 2.75 MPa of GP1-R25 to 1.75 MPa of GP1-R100 at 28 days and from 3.12 MPa to 1.98 MPa for the same mixes at 90 days. The results are similar to previous studies and reveal the fact that RCA without treatment has no effect on the tensile properties of the concrete^31–33^. Similarly in Group-2 (GP2) having slurry coating RCA exhibited slightly more improved tensile strength in comparison with GP1 meaning that treatment can help decrease some of the impacts of RCA incorporation. GP2-R25 and GP2-R100 have achieved tensile strengths of 2.55 MPa, and 1.68 MPa at 7 days, respectively. The tensile strength for GP2-R25 and GP2-R100 increases to 3.24 MPa and 2.21 MPa, respectively at 28 days. While after 90 days, the respective mixes achieved 4.52 MPa and 2.49 MPa tensile strength. In Group 2 (GP2), tensile strength was greatly improved, with a 12% improvement in concrete observed with 100% RCA, compared to 100% RCA untreated mixes. This advancement shows how slurry-coated RAC is highly effective in overcoming the incompatibility of RCA with the inherent deficiencies of RCA (adhered mortar and high porosity), these deficiencies usually compromise the ITZ integrity. The slurry coating was found to provide a cohesive layer that surrounds the RCA particles and helps in improving the ITZ, which helps in improving the bonding between particles and reduction of microstructural flaws resulting in overall improvement in tensile properties^34,35^.

Group-3 (GP3), having combo treated (1st mechanically grinded and then coated with slurry (FA + SF)) RCA have achieved the highest tensile strength; therefore, this is the best strategy appears to be more appropriate to resolve RCA related issues. Findings show that GP3-R25 and GP3-R100 developed tensile strengths of 2.81 MPa and 1.81 MPa at 7 days, respectively. While in 28 days, the values for these parameters found to be 3.58 MPa and 2.33 MPa, respectively. Similarly, after 90 days of curing, the tensile strengths of GP3R25 and GP3R100 respectively increased to 4.11 MPa and 2.61 MPa, which indicates the benefit of slurry treatment on the RAC mechanical properties. The statistical values are shown in Table 9.

The increase in pozzolanic reactivity led to improving the interfacial bond through less porous interfacial transition zone and better locking of both the aggregate and mortar in comparison with the untreated RAC. Furthermore, as fillers of the RCA, the treated materials directly entered the pores or voids of the attached mortar, thus reducing their void and pore content^36^. It could be therefore stated that the development of tensile strength is highly related to the bonding strength between the aggregative and the cemented matrix. Similar findings are reported by other research that RCA treated with pozzolan slurries improve the formation of tensile strength which can be ascribed to the formation of protective coating over the RCA and strengthening of attached mortar. Some works have shown that tensile strength of treated recycled aggregate concrete is almost like normal aggregate concrete for different curing ages^15,37–39^.

**Table 9:** Statistical values for tensile strength.

Curing days	Mean	Standard Deviation	SE of Mean
14-Days	2.16	0.48	0.13
28-Days	2.71	0.57	0.15
90-Days	2.95	0.63	0.17

### Water absorption

**Fig. 5:**
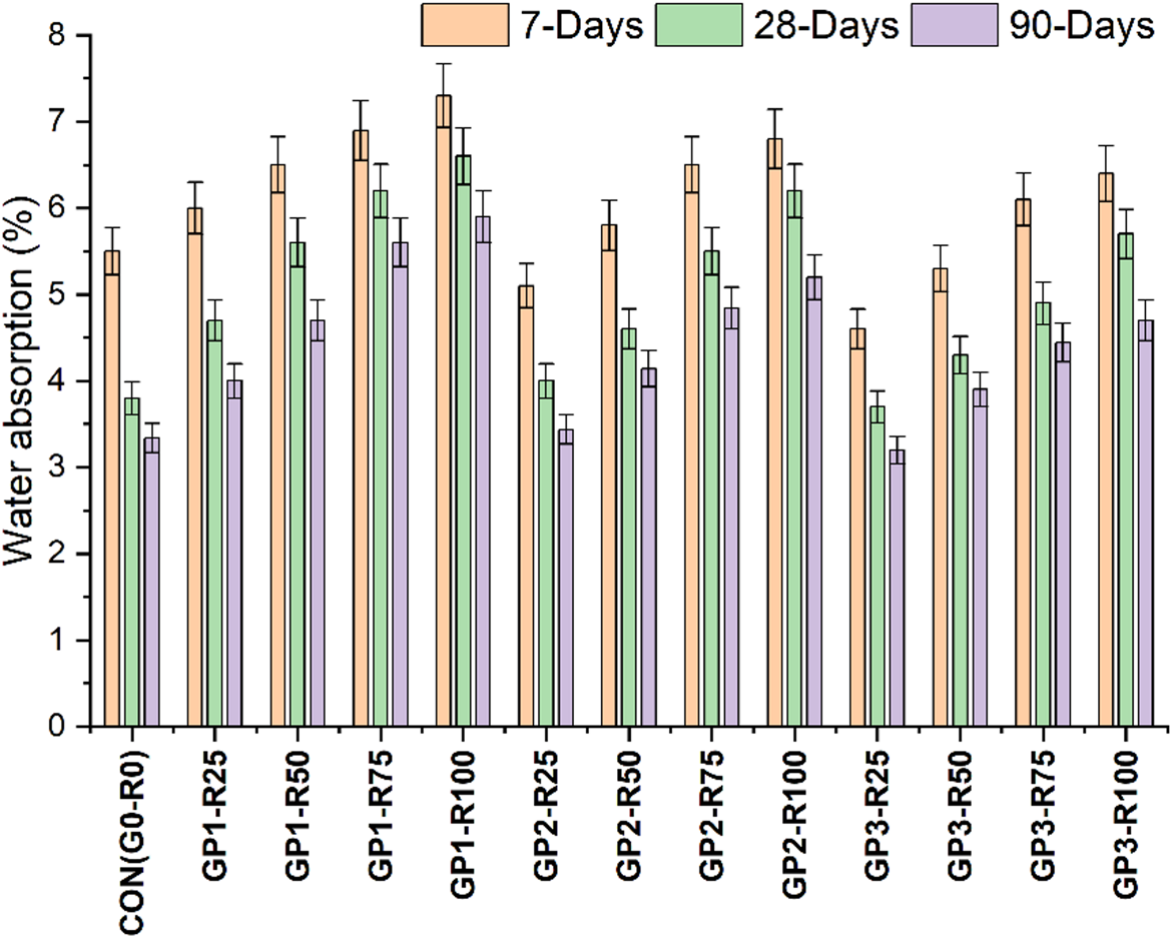
Water absorption findings of different Groups.

The findings of water absorption shown in Fig. 5 highlight the impact of untreated and treated RCA on the durability of concrete. The result indicates that treated RCA in GP2 and GP3, exhibit decrease water absorption compared to untreated RCA in GP1, suggesting enhancing durability and dense concrete matrix due to the treatment techniques. The control mix (CON-G0-R0), containing natural aggregates, showed the lowest water absorption rates, with 5.5%, 3.8% and 3.3% at ages of 7, 28 and 90 days, respectively. The results of this investigation confirm that natural aggregates have superior density and minimal porosity which produce a more durable concrete matrix with less permeability. However, Group 1 (GP1) which utilize untreated RCA revealed higher water absorption rates than the control, with an increase observed at a higher RCA content. For instance, GP1–R100 performed the highest absorption, 7.3% (7 days), 6.6% (28 days) and 5.9% (90 days). These results are attributed to the relatively high porosity and the adhered mortar surrounding the RCA, both of which contribute to the ITZ and demonstrate the deleterious impact of untreated RCA^40,41^.

The slurry coated RCA in Group 2 (GP2) absorbed less water than Group 1 (GP1), showing the sealing ability of this treatment to sealed pores and result in strong ITZ. The absorption rates at 7 days, 28 days and 90 days for GP2-R100 were found to be 6.8%, 6.2% and 5.2%, respectively, all lower than absorption rates of GP1-R100. The slurry coating reduced the aggregate porosity, resulting in significantly mitigating the negative effects of RCA, especially at higher curing ages. However, improvements were seen in Group 3 (GP3), which was coated with slurry and mechanically ground via RCA. Overall, the combo treatment resulted in the lowest levels of water absorption of the treated groups, with GP3 mixing consistently having the lowest water absorption among them. GP3-R100 had absorption values of 6.4% at 7 days, 5.7% at 28 days, and 4.7% at 90 days, indicating a substantial reduction in absorption versus GP1 and GP2. The statistical values are presented in Table 10.

The highest amount of water absorption occurred when RCA in GP1 was untreated, compromising the concrete resistance to water ingress and long-term durability. The slurry coating in GP2 slightly improved the water resistance, with GP3, the combined treatment, being most effective in improving the water resistance in improving the ITZ and reducing the porosity. These findings, finally, show that for RCA-based concrete, advanced treatment methods are necessary to achieve the best combinations of sustainable and durability enhancements for structural applications.

Similar findings showed that slurry mixtures can reduce water absorption by depending on aggregate size^42,43^. Pozzolanic materials can significantly enhance durability by decreasing void size in hydrated cement and filling up surface pores of RCAs, leading to lower permeability^43–45^. Results from slurry-treated RCA show that it can improve durability and reduce water absorption by up to 14–22% depending on the mix composition and used treatment method. Besides coating the aggregates, silica fume slurry (SFS) introduces reactive particles to the RCA’s weak zones, improving its structure. The combination of sealing surface pores and filling internal voids significantly reduces water absorption and enhances the long-term behavior of RAC. Slurry treatment also improves workability, prevents premature water absorption during mixing and leads to better slump and uniformity for fresh concrete. From these findings, it is clear how essential slurry treatments are in achieving a reduction of RCA’s inherent weaknesses and overall strengthening of mechanical properties for use in sustainable concrete applications.

The creation of a denser or more impermeable layer on the aggregate can lead to less water absorption of recycled aggregate concrete (RAC) due to slurry treatment of the recycled concrete aggregates (RCA). The slurry coating usually pozzolanic or silica rich materials, seals surface pores and reduces the porosity of adhered mortar. This treatment will infill voids and micro-cracks, when present, in the RCA to improve the surface texture and reduce its ability to hold water. Furthermore, the pozzolanic materials within the slurry react with the calcium hydroxide contained within the RCA during hydration, to form C–S–H (calcium silicate hydrate), further densifying the ITZ and reducing the overall permeability^46^.

**Table 10:** Statistical values for water absorption.

Curing days	Mean	Standard Deviation	SE of Mean
14-Days	6.06	0.78	0.21
28-Days	5.06	0.97	0.26
90-Days	4.41	0.84	0.23

### Acid resistance

**Fig. 6:**
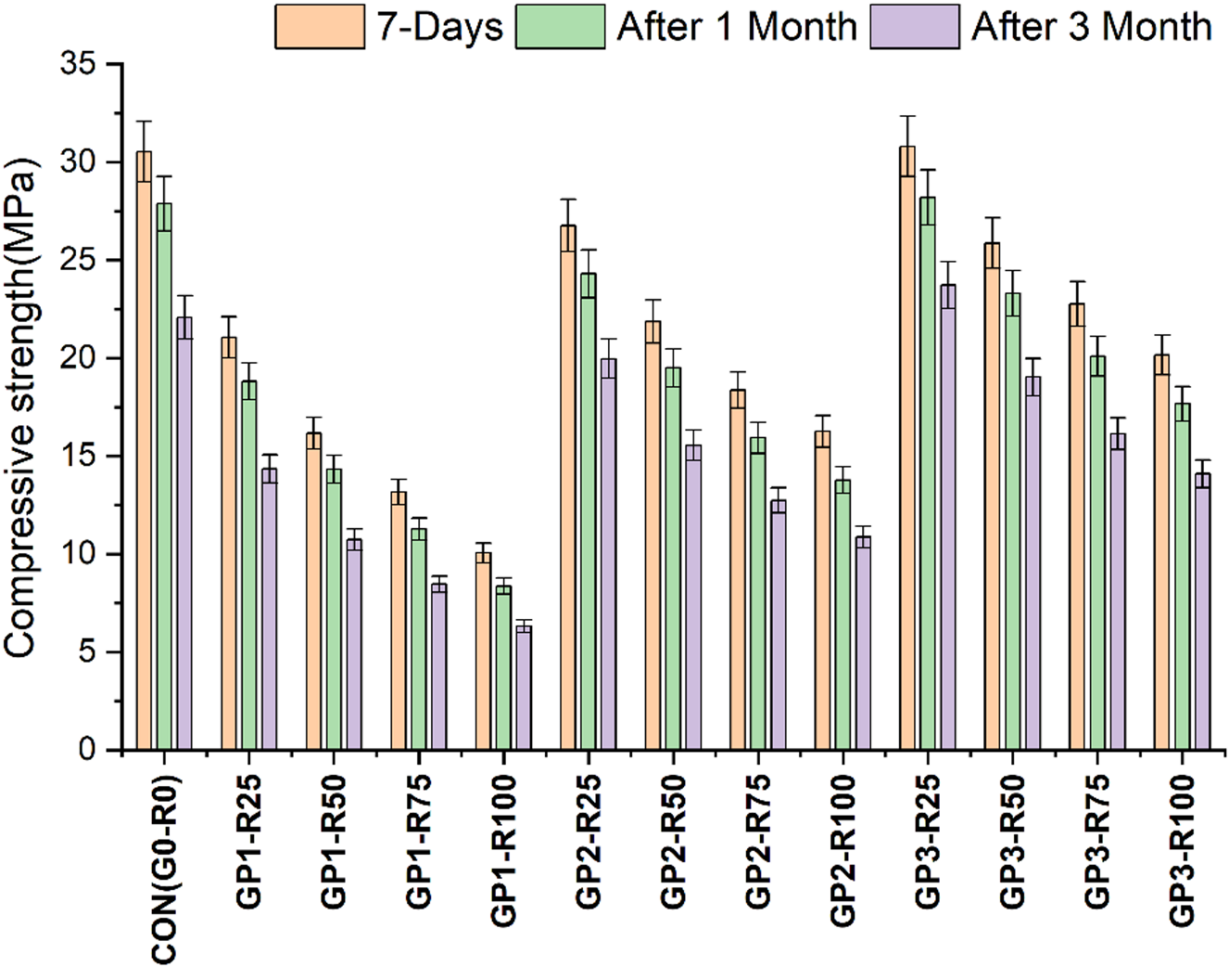
Acid attack findings of different Groups.

The concrete performance of different mixes subjected to acidic environments highlights the critical role of treatments in enhancing the durability of RAC. After an initial curing period of 7 days in water, the concrete samples were exposed to a 5% H₂SO₄ solution for 1 month and then for 3 months. The strength retention and acid resistance of RCA-based concrete is determined in this process, resulting in valuable knowledge about its long-term performance in these environments. The results shown in Fig. 6 reveal that for control samples (CON(GORO)) the initial strength 30.53 MPa decreased moderately to 27.87 MPa after 1 month and later to 22.07 MPa after 3 months exposure. This corresponds to strength retention of 91.3% after one month and 72.3% after three months.

In Group 1, the untreated RCA showed significant vulnerability to acid exposure, which is observed, with strength losses proportional to content of RCA. For GP1-R100, with 100% RCA, showed a drop in strength from 10.06 MPa at 7 days to 8.37 MPa after 1 month which is 16% lower similarly to the strength recorded 6.33 MPa after 3 months, maintaining only 62.9% of its initial strength. Untreated RCA contains a high porosity and a weak interfacial transition zone (ITZ) and permits acids to penetrate deeply to dissolve adhered mortar and potentially compromise structural integrity^47^.

However, there was a better resistance to acidity in slurry coated RCA (Group 2). The incorporation of FA and SF slurry plays the role of coating the RCA, blocking the pores and hence minimizing the permeability. This treatment greatly improved the RAC’s durability by reinforcing the ITZ and minimizing the risk of acid infiltration. In case of GP2-R100 result shows that strength decrease from 16.26 MPa at 7 days up to 13.77 MPa after 1 month and 10.86 MPa after 3 months, meaning the strength retention of 66.8%. Slurry-coated mixes lost strength more slowly than untreated RCA, GP2-R25 retained 74.6% of its initial compressive strength after 3 months, which was higher than in GP1-R25.

The treatment for RCA as Group 3 (mechanical grinding treated with coating of slurry) showed maximum resistance to acid attack than any other RCA mix. The combo treatment of adhered mortar removal and aggregate surface sealing achieved a dense and robust ITZ. For example, GP3-R100, which was aged for 3 months, lost only 30.1% of the initial strength (69.9%), which was considerably better than that of untreated as well as slurry coated mixes. One of the notable aspects is that GP3R25 showed great durability with the retention of 77% of its initial strength (30.80 MPa at 7 days) after 3 months of exposure, this is very close to that of the control mix. Results show a definite trend of strength reduction in acidic conditions, with untreated RCA being the most vulnerable due to its porous and weak microstructure. These weaknesses are significantly mitigated by the application of slurry coatings and the combined treatment approach provides the best solution for improving acid resistance. This shows that advanced treatment methods such as RCA should be developed to improve its durability in chemical environments so to broaden its applications in sustainable construction. The statistical values of all groups are shown in Table 11.

RCA Microstructural and durability properties are enhanced significantly by the treatment methods, which are greatly reflected in their acid resistance. By means of slurry coatings, silica fume (SF) layer imparts good acid resistance to the RCA surface layer by making it denser and less permeable^7^. Pozzolanic reaction between SF and calcium hydroxide (Ca (OH)₂), a byproduct of cement hydration, can form additional C-S-H (calcium silicate hydrate), which can improve performance. C-S-H generation strengthens the ITZ and lowers porosity, which effectively seals the weak areas found in the adhered mortar and aggregates^47^.

Slurry coatings have significantly reduced permeability through microstructural enhancements. Recycled aggregates and new cement paste form dense ITZ, the form of a dense ITZ around the recycled aggregates and new cement paste further reduces the acid penetration and enhances the acid attack resistance of the recycled aggregate concrete by reduction of the ingress of aggressive substances^48^. In addition, SF particles fill up micro and macro voids in the aggregates and adhered mortar, and possess acid resistance, which significantly enhances the acid-resistant properties of concrete and thus prevent penetration of acid and mitigate its destructive effects. The acid attack susceptibility of the formed products, especially those containing Ca (OH)₂, is therefore effectively reduced by this process, and more stable hydration products like C-S-H are formed^44,49^.

Several studies show that the treatment of RCA with slurry coating greatly reduces the degradation resulting from acidic environments^27,50,51^. This method enables protection of aggregates and concrete matrix from acid access due to decrease porosity and the denser microstructure, which improves the lifespan of recycled aggregate concrete (RAC). As a result, the incorporation of slurry coated RCA is a viable path forward for increasing acid resistance, compatible with sustainable construction practices, and strengthens the durability of concrete in destructive environments.

**Table 11:** Statistical values for acid attack.

Curing days	Mean	Standard Deviation	SE of Mean
14-Days	21.06	6.33	1.75
28-Days	18.72	6.07	1.68
90-Days	14.92	5.22	1.44

### UPV technique

**Fig. 7:**
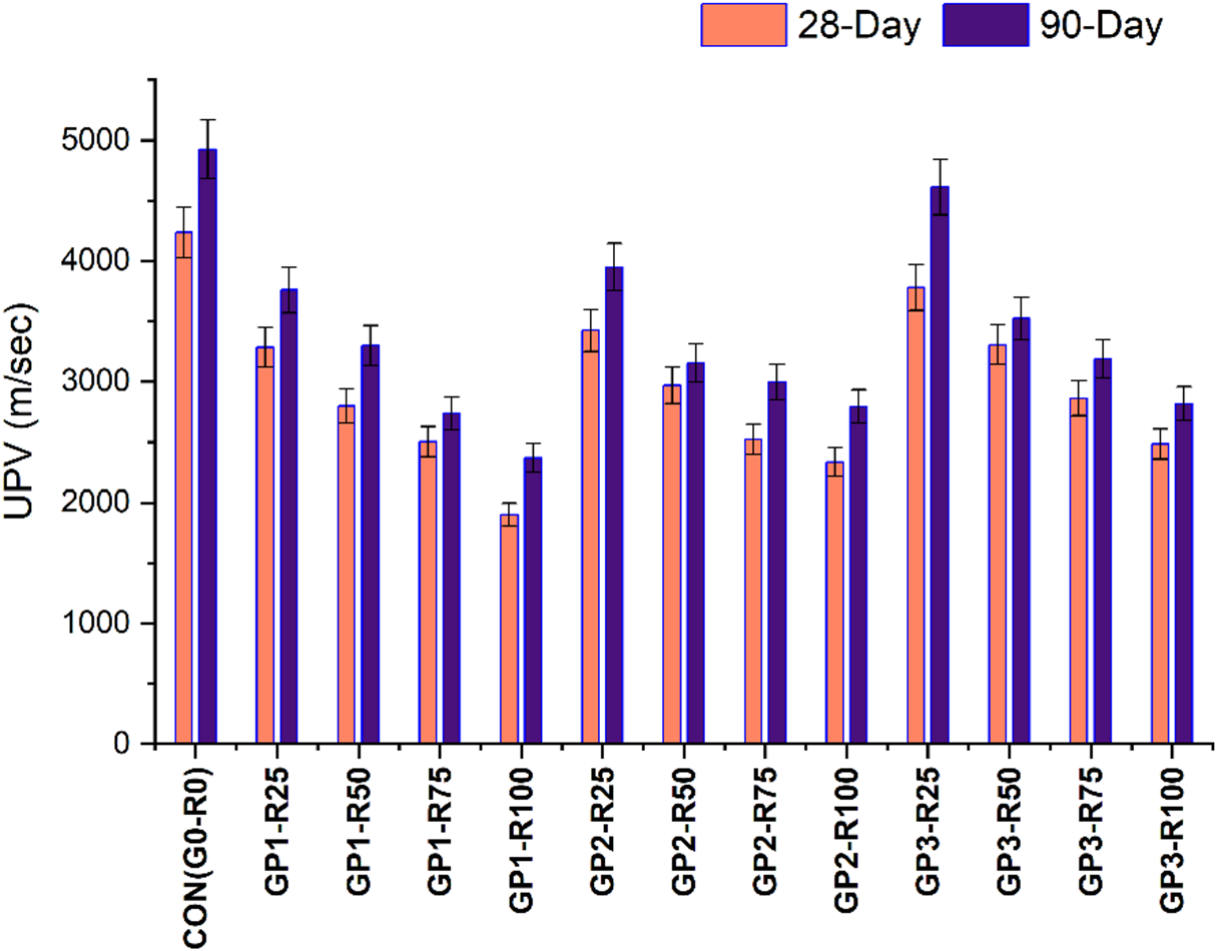
UPV findings of different Groups.

The UPV (Ultrasonic Pulse Velocity) is proportional to the shear modulus, bulk modulus and density of the material and is therefore a good indicator of concrete’s characteristics. As porosity and cracking have a direct impact on these properties, they greatly impact UPV readings^52^. Many cracks in the material or numerous pores decrease the possibility for the ultrasonic wave, and thus lower UPV values are obtained^53,54^.The UPV findings showed in Fig. 7 at 28 and 90 days in the concrete mixes showed some of the quality, homogeneity and durability of the various mixes. At 28 days 4234.56 m/s and at 90 days 4925.58 m/s, the control mix (CON-G0-R0) consistently possesses the highest UPV values, which may indicate that this control mix possesses better concrete quality, with high density and low porosity. The rise of UPV with time demonstrates further hydration and densification of the concrete matrix and hence, tends to enhance the durability and integrity of the matrix. However, the UPV values of the untreated RCA mixes (Group 1) are noticeably lower compared to the control mix and follow the trend of declining UPV with increasing RCA content. For instance, GP1-R25 exhibits 3284.16 m/s at 28 days and 3758.7 m/s at 90 days, whereas GP1-R100 shows the lowest m/s of 1900.8 m/s at 28 days and 2367.42 m/s at 90 days. Weak interfacial transition zone (ITZ), high porosity and the existence of adhered mortar in RCA are responsible for the reduced UPV in untreated RCA mixes as they influence homogeneity and continuity in the concrete matrix^36,55^. The increased RCA content leads to lower UPV, which corresponds to its poor effect on concrete quality^56^.

The fact that the UPV values for the slurry coated RCA mix (Group 2) differ positively from the ones for the untreated RCA mix shows the effect of the slurry treatment as being reduction in porosity and an increase in the strength of the interfacial transition zone. This is also because GP2 − R25 shows values of 3421.44 m/s at 28 and 3949.44 m/s at 90 days which are significantly higher than GP1-R25. Even at higher RCA contents such GP2-R100, the UPV values (2333.76 m/s at 28 days and 2793.78 m/s at 90 days) show an improvement when compared with the corresponding untreated mix (GP1-R100). These results suggest that slurry coating could be applied for the enhancement of homogeneity and densification of RCA-based concrete, ensuring material durability and robustness against severe conditions.

This study demonstrates GP3-R25 has increased UPV to 3780.48 m/s at 28 and then 4611.42 m/s at a 90-day mark. The porosity of the surface pores has been decreased through a new and effective method for removing sealing mortar or grinding and combining those materials.

The improvements in the UPV values for the slurry coated RCA mixes (Group 2) compared to the untreated RCA mixes indicate that slurry treatment reduced porosity and increased the strength of the ITZ. It can also be seen that GP2-R25 records 3421.44 m/s at 28 days and 3949.44 m/s at 90 days, which are much higher than GP1-R25. At higher RCA contents, such as GP2-R100, the UPV values (2333.76 m/s at 28 days and 2793.78 m/s at 90 days) still show an improvement in comparison to the corresponding untreated mix (GP1-R100). These results indicate that slurry coatings can be used to improve the homogeneity and density of RCA-based concrete, making it more durable and more resistant to aggressive environments. Mixes specifically treated with the combination of treatments (Group 3) have the highest UPV values among the RCA-based groups. Results show that GP3-R25 achieved 3780.48 m/s at 28 days and 4611.42 m/s at 90 days equivalent to a significant increase in the concrete quality. Furthermore, at the highest level of RCA, GP3-R100 shows 2481.6 m/s at 28 days and 2816.22 m/s at 90 days, achieving higher values than those reached by the related GP1 and GP2 mixes. An effective combined method for removing adhered mortar and sealing surface pores is mechanical grinding and slurry coating, making concrete denser and more homogenous. This dual treatment improves substantially the quality and the durability of RCA-based concrete. The statistical values are shown in Table 12.

The UPV values tend to reduce with the rise of the RCA content in all the groups, especially in the untreated RCA due to its poor quality and highly porous structure. Most of the treatments, for instance, slurry coatings and combo treatments greatly enhance UPV and therefore reduce porosity and strengthen the ITZ^57^. Combo treatment is the most effecti ve according to the evaluation indicating that it addresses the shortcomings of RCA. The control UPV values rise from 28 to 90 days for all mixes due to rising stiffness of the concrete structure over time. Comparing the values of treated and untreated mixes, the latter demonstrates more significant changes suggesting enhanced long-term performance. Incorporation of untreated RCA in concrete lowers concrete quality as characterized by its porous structure and inferior ITZ as projected in the UPV results. However, other techniques like slurry coating and both mechanical and chemical method make the RCA-based concrete perform better^27,48^.

**Table 12:** Statistical values for UPV.

Curing days	Mean	Standard Deviation	SE of Mean
28-Days	2953.55	639.82	177.45
90-Days	3393.61	749.09	207.76

### Rebound number

**Fig. 8:**
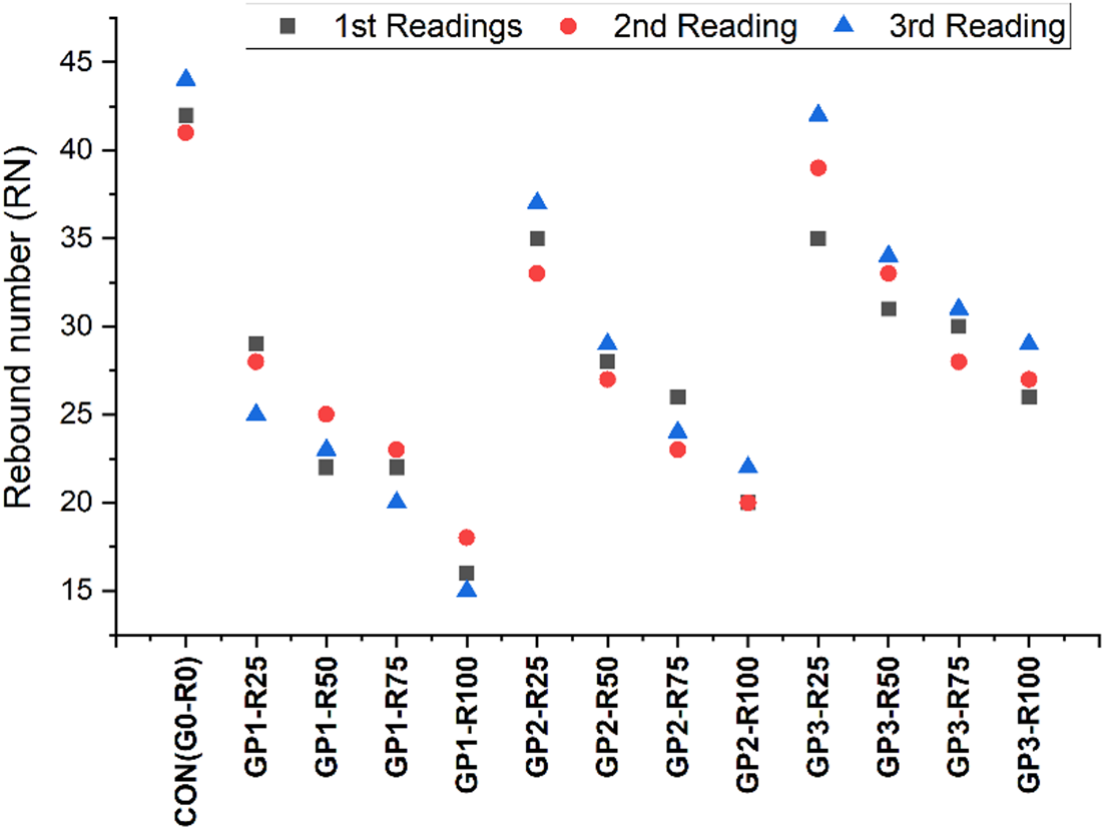
RN findings of different Groups.

Figure 8 shows 28-day compressive strength and corresponding rebound number (RN) of various concrete mixtures considering the influence of RCA proportion and its treatments on mechanical and surface characteristics. From the control mix (CON-G0-R0), it has been observed that the compressive strength is 43.97 MPa which resembles that the surface hardness, density and structure are superior to the others with RN value of 42, 41 and 44 respectively. This performance is attributed to the fact that natural aggregates yield a denser and more homogeneous concrete matrix with a better ITZ^58^.

Group 1 (GP1 – untreated RCA) shown a decrease in RN values, as the percentage of RCA replacement increases. For instance, GP1-R25 sample has compressive strength of 30.5 MPa and RN values of 29, 28 and 25 while the lowest strength belongs to GP1-R100 with 18.21 MPa and RN of 16, 18 and 15 respectively. These reductions can be attributed to the weaker ITZ and the higher porosity value in the untreated RCA, because the porous adhered mortar in RCA destroys the matrix continuity and surface hardness and thus brings much variability in RN value. Group 2 (GP2 - slurry-coated RCA) experienced enhanced performances compared to the RCA mixes without coatings. The samples of GP2-R25 provide a compressive strength of 36.51 MPa and RN values of 35,33,37 more than GP1-R25. As observed, compared to untreated mixes, both the compressive strength and RN values at higher RCA contents; that is, GP2-R100, are impressive; that is, 24.46 MPa and 20,20,22, respectively. The slurry coating decreases the porosity of the RCA surface, enhances the ITZ and leads in the RN values and the mechanical properties of the composites. The statistical value for each group is shown in Table 13.

Among the RCA based mixes, GP3 (combo-treated RCA) shows relatively the best performance as far as compressive strength and RN values are considered and are quite closer to those of the control mix. GP3-R25 results in a compressive strength of 40.4 MPa and RN values of 35,39,42 show that mechanical grinding together with slurry coating improves the quality of aggregate and surface hardness. At higher RCA contents such as (GP3-R100) the compressive strength is 27.49 MPa and RN values 26,27,29 are much higher than for GP1-R100 and GP2-R100 indicating the effectiveness of combined treatments on homogeneity and durability of RCA based concrete. As observed from the trends, both the compressive strength and RN values reduce with the increment in the RCA content for all the groups. The mechanical grinding and the slurry coating in GP3 give the most consistent and durable results to make it the most successful way to enhance RCA based concrete for structures. Thus, these results indicate the role of RCA treatments to improve not only structural strength through compressive strength but also the superior surface hardness needed for the development of structures that are long-lasting and eco-friendly.

**Table 13:** Statistical values for rebound number.

Curing days	Mean	Standard Deviation	SE of Mean
1 st Reading	27.84	7.04	1.95
2nd Reading	28.07	6.86	1.90
3rd Reading	28.84	8.61	2.38

## Microstructure characterization

**Fig. 9:**
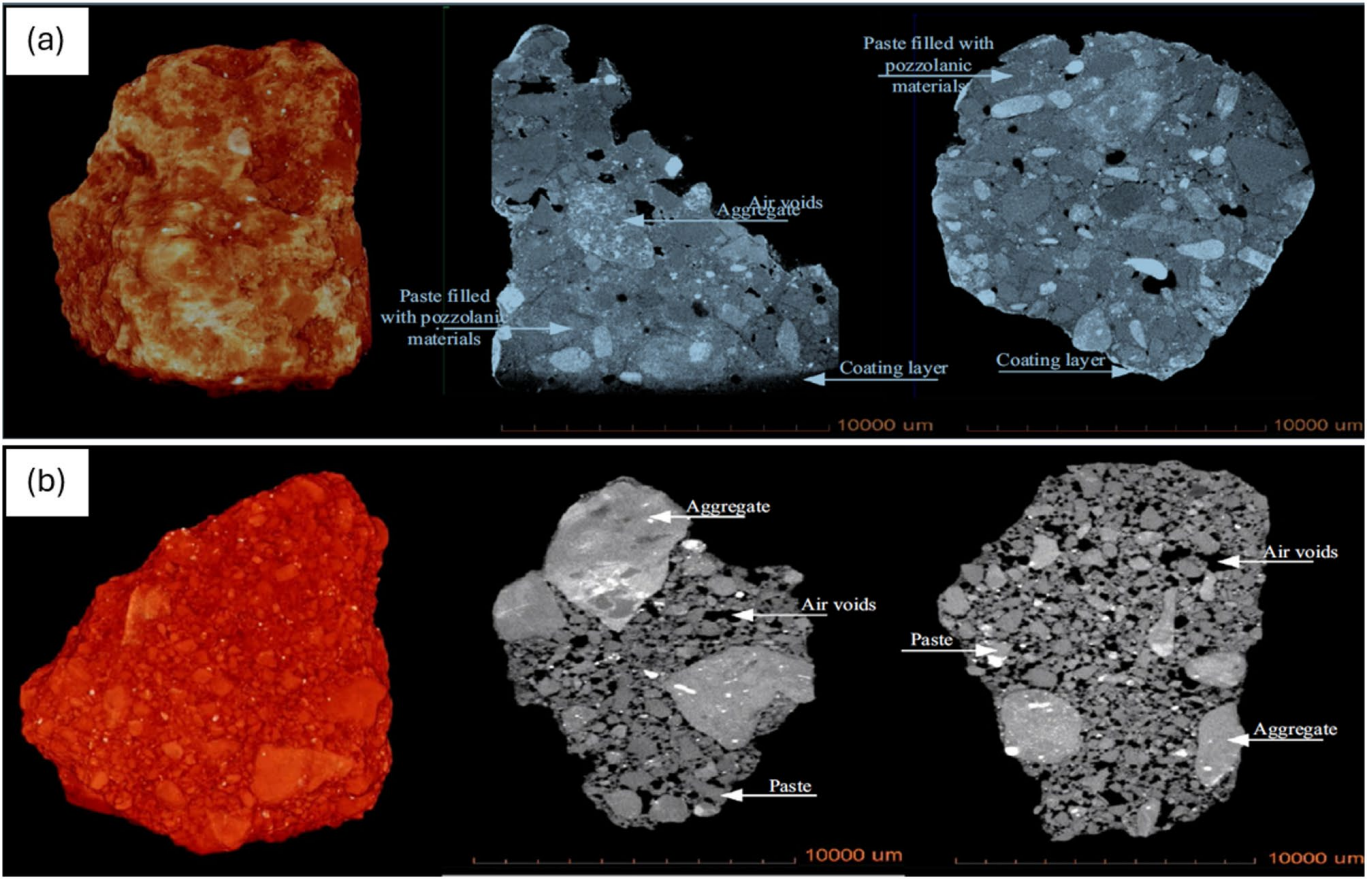
X-µCT images (**a**) Untreated RA (**b**) Treated RA with slurry^23^.

Based on previous study^23^, the outcomes of the present X-µCT (X-ray micro computed tomography) analysis correspond to the existing knowledge regarding the impact of treatment on RCA (Fig. 9). The untreated RCA presents invariably with highly porous structure where porosity is manifested by irregular air voids, features due to adherent mortar. The research has established that this porosity reduces the mechanical characteristics and durability of RCA-based concrete. However, treatments with pozzolan slurries, as illustrated in the X-lCT images above, prevent the occurrence of high porosity and air voids since these slurries fill up microstructural voids and consolidate the surface of aggregates. Past studies substantiate that these treatments improve the ITZ, advancing aggregate characteristics, minimizing permeability, and increasing mechanical and durability performance of concrete. These findings support slurry treatments’ eligibility as a dependable approach to address the challenges of untreated RCA in the context of sustainable and high-performing recycled aggregate concrete.

SEM analysis carried out in Previous studies have pointed out that surface treatment techniques significantly enhance the interfacial transition zone (ITZ) and old mortar in recycled aggregate concrete (RAC). Mortar surface appears to have porous areas, micro cracks and CH crystals are accumulated in voids and mortar surface. A weak ITZ structure can be observed with untreated RAC, and this has adverse impacts on the strength and durability of RAC. It has been demonstrated, however, that the application of pozzolanic materials in surface treatments significantly addresses these constraints.

**Fig. 10:**
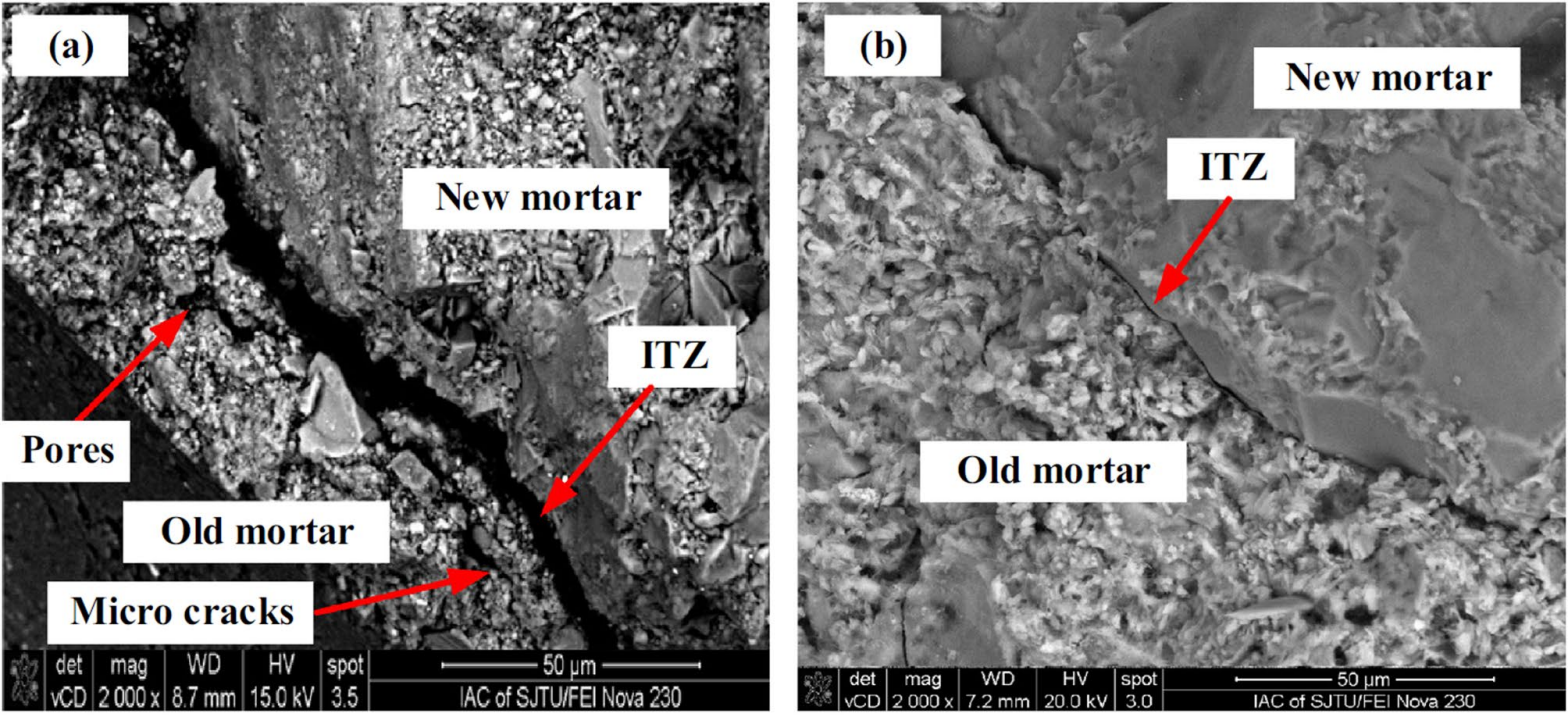
SEM analysis (**a**) Untreated RAC (**b**) Treated RAC with Slurry^23^.

SEM images shown in Fig. 10 confirm the visual differences in microstructure of interfacial transition zone (ITZ) and old mortar in untreated and treated recycled aggregate concrete (RAC). Untreated RAC has a highly porous ITZ with prominent microcracks and voids in old mortar wherein the bond between the new and old cement mortar is compromised, as shown in Image (a). The porous adhered mortar on the RCA is the cause of the weak ITZ that significantly affects the mechanical and durability properties of the concrete. Conversely, image (b) shows the densified ITZ in treated RAC, which demonstrates that the surface treatment has reduced porosity and sealed cracks of the old mortar. This identifies the impact of pozzolanic materials like silica fume and nano silica where both react with calcium hydroxide to form C–S–H and bond better to the RCA. In this reaction, voids and cracks in microstructure are filled and microstructure is made stronger and denser.

## Conclusion

- The combination of 1% PP fibers, 30 kg/m³ silica fume (SF), and 60 kg/m³ metakaolin (MK) significantly improved the mechanical and durability properties of recycled coarse aggregate concrete (RAC). Compressive strength increased by 18%, tensile strength increased by 48% compared to control mix, reaching 6.9 MPa at 180 days.
- The use of pozzolanic materials (SF and MK) in recycled aggregate concrete significantly reduced water absorption by 28%, primarily through pore refinement and ITZ densification, which effectively sealed microcracks in the adhered mortar and created a more impermeable concrete matrix.
- The treated RAC showed excellent acid resistance, losing 40% less strength than untreated concrete when exposed to acids. SF and MK created a denser, less permeable structure that blocked acid penetration, while forming extra C-S-H gel to strengthen the concrete against chemical exposure.
- The treated RAC showed 15–20% higher UPV and 25% better rebound values than untreated mixes, achieving near-NAC performance (within 10%) due to pozzolan-induced ITZ densification and 30–35% porosity reduction.

Finally, surface treatment techniques are beneficial to increase RAC performance. These treatments improve mechanical properties; that is, compressive and tensile strengths, and reduce water absorption and increase acid attack resistance. Better density and homogeneity are shown with higher Ultrasonic Pulse Velocity (UPV) and Resistivity Number (RN) values. However, several limitations exist in this study, and further research is needed to address them. One key area for future exploration is the long-term aging effects of treated RCA in concrete, as the durability benefits observed in this study should be assessed over extended periods to ensure long-term performance. Additionally, the field implementation of these treatments presents challenges, such as the scalability of slurry coating and the energy consumption associated with mechanical grinding. These practical considerations must be examined to determine the feasibility of large-scale adoption in real-world construction projects. Finally, further studies should explore additional concrete properties, such as shrinkage and thermal conductivity, to provide a more comprehensive understanding of the full range of performance characteristics of treated RCA. Addressing these areas will help enhance the applicability and sustainability of RCA-based concrete in the construction industry.

## Data Availability

The data is available from the corresponding author upon request.
